# Case Report: Percutaneous portal-central venous bypass: a novel salvage therapy for refractory variceal bleeding in TIPS-ineligible patients with portal vein tumor thrombus

**DOI:** 10.3389/fmed.2026.1731912

**Published:** 2026-02-02

**Authors:** Guoyu Deng, Shaomei Tang, Meiling He, Chaonan Zhou, Xiaoju Chen

**Affiliations:** 1 Department of Interventional Radiology, Guangxi Medical University Cancer Hospital, Nanning, Guangxi, China; 2 Department of Internal Medicine, Guangxi Medical University Cancer Hospital, Nanning, Guangxi, China; 3 Department of Day Oncology Unit, Guangxi Medical University Cancer Hospital, Nanning, Guangxi, China; 4 Guangxi Key Laboratory of Extremely Weak Magnetic Field in Cancer Medicine, Guangxi Medical University Cancer Hospital, Nanning, Guangxi, China

**Keywords:** acute variceal bleeding, hepatocellular carcinoma, percutaneous portal-central venous bypass (PPCV), portal vein tumor thrombus, salvage therapy

## Abstract

**Background:**

Acute esophagogastric variceal bleeding (EGVB) is a lethal complication in patients with hepatocellular carcinoma (HCC) complicated by portal vein tumor thrombus (PVTT). While transjugular intrahepatic portosystemic shunt (TIPS) is a standard treatment for portal hypertension, it is often contraindicated or technically challenging in patients with extensive main portal vein occlusion (Vp4 type) or severe hepatic dysfunction.

**Case summary:**

We report the first clinical application of a novel salvage technique, percutaneous portal-central venous bypass (PPCV), in a 56-year-old man with HCC, decompensated cirrhosis, and extensive PVTT. The patient presented with life-threatening EGVB refractory to endoscopic therapy and was deemed ineligible for TIPS due to the extent of the tumor thrombus and critical instability. PPCV was performed by percutaneously puncturing the portal vein under ultrasound guidance and establishing an extracorporeal shunt connected to a pre-existing subclavian central venous catheter.

**Results:**

The procedure was technically successful and achieved immediate hemodynamic improvement. Direct portal venous pressure decreased significantly from 40.6 mmHg pre-connection to 18.8 mmHg post-connection. Clinical hemostasis was achieved within 24 h, and the patient remained stable for 2 weeks until discharge. Although the patient succumbed to tumor progression 2 months later, no recurrent bleeding or procedure-related complications were observed.

**Conclusion:**

PPCV is a simple, feasible, and effective salvage therapy for rapidly reducing portal pressure and controlling refractory bleeding. It provides a vital therapeutic option for high-risk HCC patients with PVTT who are contraindicated for TIPS.

## Introduction

Hepatocellular carcinoma (HCC) is one of the most common malignancies worldwide and a leading cause of cancer-related mortality (1–4). It is frequently associated with liver cirrhosis and portal hypertension (PH). Notably, at the time of diagnosis, approximately 10–40% of patients already present with portal vein tumor thrombus (PVTT) (5, 6). PVTT not only obstructs portal venous flow, exacerbating portal hypertension, but is also significantly associated with a poor prognosis. It leads to complications of symptomatic portal hypertension (SPH), such as variceal bleeding, refractory ascites or hydrothorax, portal hypertensive enteropathy (PHE), and diarrhea (7–9).

In China, approximately 30–40% of HCC patients present with concurrent PVTT (10). The presence of PVTT further aggravates portal hypertension and deteriorates liver function, significantly increasing the risk of acute esophagogastric variceal bleeding (EGVB). EGVB remains a direct cause of death in many HCC patients, particularly those with decompensated cirrhosis (11). For HCC patients with PVTT, the median overall survival (OS) with conservative treatment is merely 2.7 months (12, 13).

In recent years, with the advent of targeted therapy and immunotherapy, the prognosis for patients with Barcelona Clinic Liver Cancer (BCLC) stage C HCC has significantly improved, with the median OS increasing from 2.7 months to 19.2 months (12, 14). However, notably, the incidence of EGVB has concurrently risen (15). Endoscopic therapy combined with vasoactive drugs is the mainstay of treatment for acute EGVB (16). Nevertheless, endoscopic treatment fails in 10–20% of cases, and the risk of rebleeding within 48–72 h remains high (17). When endoscopic intervention fails, non-endoscopic salvage measures, such as hemostatic drugs and shunt procedures, must be considered (16).

In this context, the transjugular intrahepatic portosystemic shunt (TIPS) has gained prominence as an effective method for managing PH complications (18, 19). However, the safety and efficacy of TIPS in treating SPH and EGVB induced by HCC with PVTT—especially involving the main portal vein (Vp4-type)—remain controversial, and the procedure is often considered contraindicated (20).

To address such complex clinical scenarios, we developed a simplified and feasible novel technique: percutaneous portal-central venous bypass (PPCV). This procedure involves ultrasound-guided percutaneous puncture of the portal vein, followed by the creation of an extracorporeal portosystemic shunt via a central venous catheter, aimed at rapidly reducing portal pressure and achieving hemostasis. In this study, we report a case of refractory EGVB successfully managed with PPCV. The patient was critically ill following failed endoscopic hemostasis and was ineligible for TIPS due to progressive liver failure and extensive main portal vein tumor thrombosis. Although the underlying malignancy ultimately claimed his life 2 months later, this innovative intervention offers a potential solution for similar high-risk patients. This case report adheres to the CAse REport guidelines.

## Case presentation

### History and admission

A 56-year-old man was admitted to our hospital on 28 October 2024 with complaints of fatigue and anorexia persisting for over 10 days, which developed following targeted immunotherapy for HCC. The patient’s medical history was significant for HCC (CNLC Stage IIIa) diagnosed in September 2024, for which he had received hepatic arterial infusion chemotherapy (HAIC) and targeted immunotherapy. He had developed melena on 17 October 2024, which reportedly resolved after local hemostatic therapy. He also had a history of chronic hepatitis B virus infection, managed with tenofovir.

**Figure 1:**
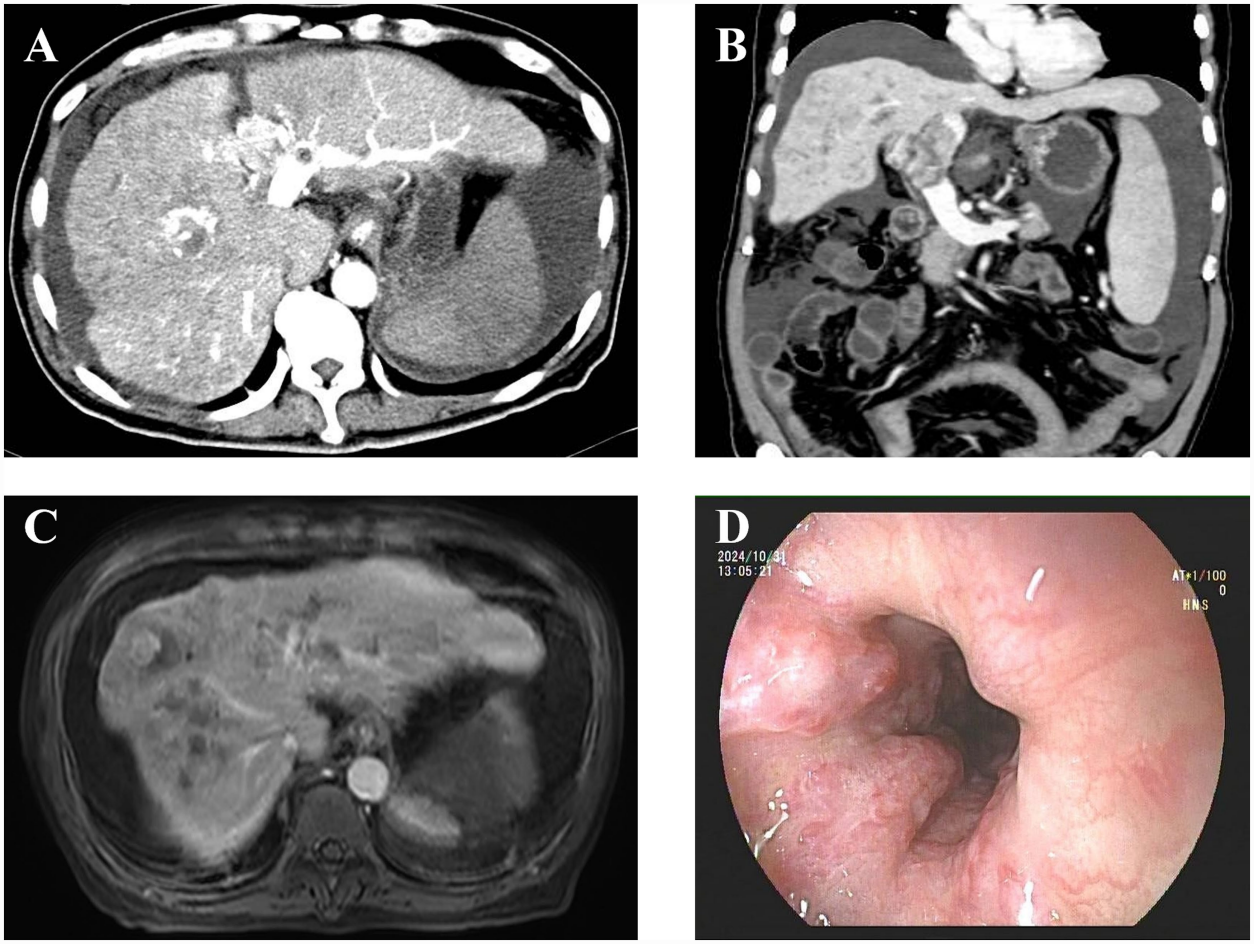
Preoperative imaging and endoscopic findings. **(A)** Axial CT showing tumor thrombus in intrahepatic portal branches (arrows). **(B)** Coronal CT demonstrating complete occlusion of the main portal vein (arrow). **(C)** MRI confirming HCC with extensive portal vein tumor thrombus (PVTT). **(D)** Endoscopy revealing severe esophagogastric varices.

On admission, vital signs were stable. Physical examination revealed mild scleral icterus and abdominal distension with shifting dullness. Laboratory assessments performed on 29 October 2024 revealed significant cytopenia and hepatic dysfunction: white blood cells (WBCs) were 9.74 × 10^9^/L, hemoglobin (Hb) was 99 g/L, and platelets (PLTs) were 136.00 × 10^9^/L. Liver function tests indicated impaired synthesis and cholestasis with total bilirubin (TB) of 47.4 μmol/L, albumin (ALB) of 31.7 g/L, alanine aminotransferase (ALT) of 57 U/L, and aspartate transaminase (AST) of 104 U/L. Coagulation profiles showed a prothrombin time (PT) of 10.8 s and an activated partial thromboplastin time (APTT) of 39.8 s. Additionally, tumor markers were markedly elevated, with alpha-fetoprotein (AFP) being negative but abnormal prothrombin (PIVKA-II) of 10148.00 ng/mL, CA125 of 607.3 U/mL, and CA19-9 of 45.9 U/mL. The patient also tested positive for hepatitis B surface antigen (2720.81 IU/mL) with a blood ammonia level of 20 μmol/L. The diagnostic workup included magnetic resonance imaging (MRI), which confirmed multiple hepatic lesions, extensive PVTT, cirrhosis, and esophagogastric varices. An enhanced computed tomography (CT) scan further detailed the PVTT extending into the hepatic veins. An upper endoscopy performed on 30 October 2024 revealed severe esophagogastric varices, which were treated with endoscopic ligation and sclerotherapy (Figure 1).

Based on these findings, the patient was diagnosed with: (1) decompensated cirrhosis with esophagogastric variceal hemorrhage, (2) hepatocellular carcinoma (CNLC Stage IIIa) with main portal vein tumor thrombus, and (3) chronic hepatitis B virus infection.

### Rescue procedure and clinical course

**Figure 2:**
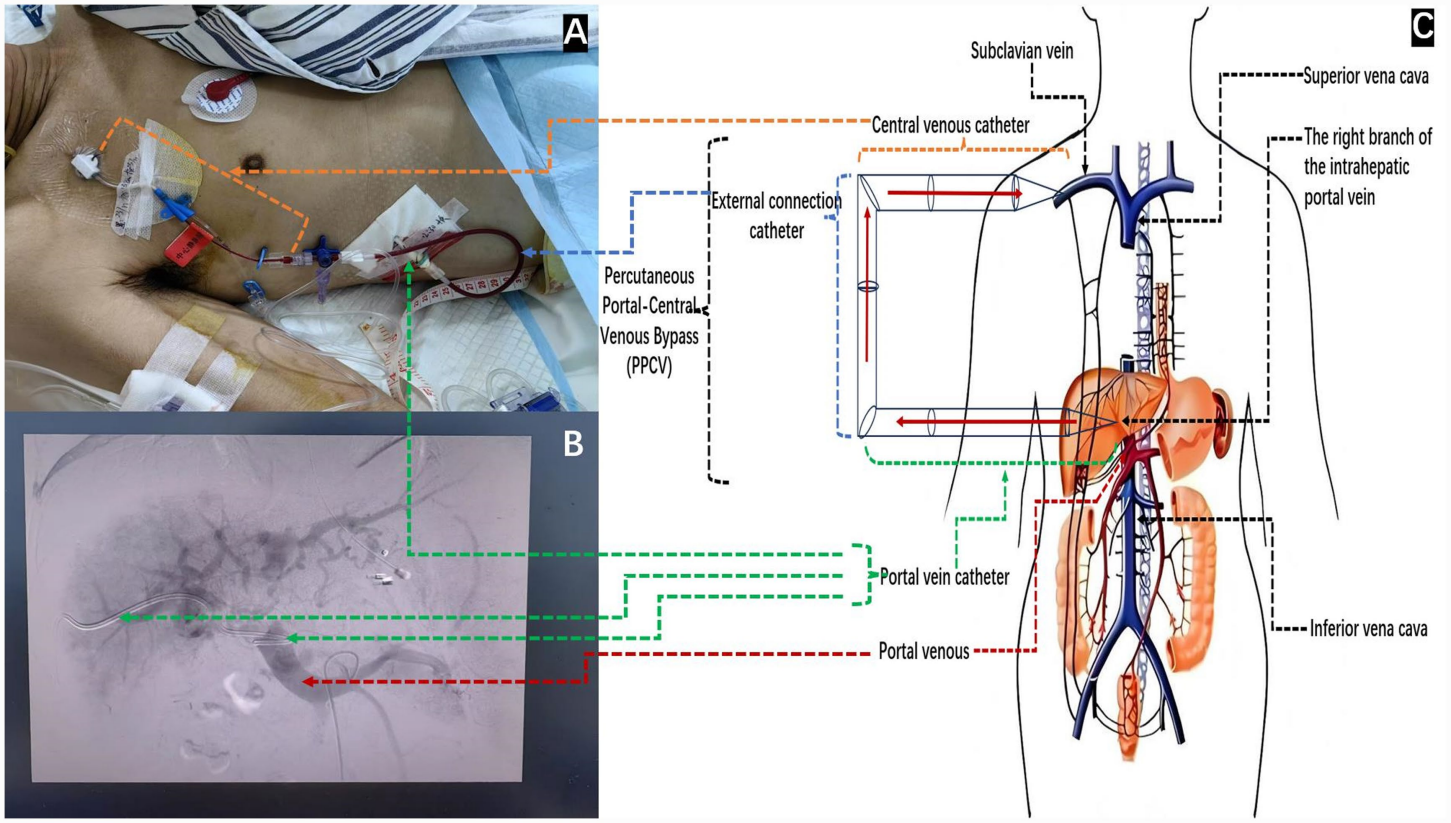
Percutaneous portal-central venous bypass (PPCV) procedure. **(A)** External setup: The portal catheter (green arrow) is connected to the central venous catheter (yellow arrow) via a connecting tube (blue arrow). **(B)** DSA image: The portal catheter tip is positioned in the superior mesenteric vein (green arrow). **(C)** Schematic: Illustration of the portal-to-systemic shunt mechanism (red arrows) reducing portal pressure.

Following admission, the patient developed hypovolemia and shock due to recurrent bleeding from the esophagogastric varices. To facilitate urgent fluid resuscitation and blood transfusion, a central venous catheter (SCW-CVCP-1, 16G × 20 cm, 1 Lumen, Shenzhen, China) had been routinely placed in the right subclavian vein in the intensive care unit (ICU) (Figure 2, yellow dashed arrow) (see Table 1).

**Table 1:** Timeline of diagnosis and treatment.

Date	Clinical event	Management and outcome
5 September 2024	Initial diagnosis	Diagnosed with HCC (CNLC Stage IIIa) with extensive PVTT and chronic HBV infection. Initiated HAIC combined with targeted immunotherapy
17 October 2024	First bleeding episode	Developed melena, treated with hemostatic agents and gastric protection. Symptoms temporarily resolved
28 October 2024	Hospital admission	Admitted for decompensated cirrhosis and severe esophagogastric varices
30 October 2024	Endoscopic intervention	Upper endoscopy confirmed severe varices. Performed endoscopic ligation and sclerotherapy (Figure 1)
18 November 2024	Life-threatening hemorrhage	Recurrent massive hematemesis and melena leading to hypovolemic shock. Transferred to ICU. Fluid resuscitation and blood transfusion via a central venous catheter
21 November 2024	PPCV procedure (Day 0)	Salvage therapy: MDT confirmed TIPS ineligibility. Performed percutaneous portal-central venous bypass (PPCV) Outcome: Portal pressure dropped from 40.6 mmHg to 18.8 mmHg immediately
22 November 2024	Post-PPCV (Day 1)	Hemostasis achieved: No active bleeding from the gastric tube; melena ceased. Continuous heparin infusion initiated to maintain catheter patency
24 November 2024	Post-PPCV (Day 3)	Diet resumption: Patient resumed a liquid diet with no recurrence of bleeding
28 November 2024	Post-PPCV (Day 7)	Ward transfer: Hemodynamics stable. Transferred from the ICU to the general internal medicine ward
5 December 2024	Post-PPCV (Day 14)	Discharge: Condition stabilized with no complications (e.g., normal blood ammonia). PPCV catheters removed. Discharged home
January 2025	Follow-up/end of clinical course	Death: The patient succumbed to multiple organ failure secondary to the progression of advanced HCC (approximately 2 months post-procedure)

Following the onset of severe EGVB and the failure of endoscopic hemostasis, the patient’s condition became critical. Given that TIPS was contraindicated due to the extensive main portal vein tumor thrombus and progressive liver dysfunction, a multidisciplinary team (MDT)—comprising internal medicine, interventional radiology, ICU, endoscopy, and ultrasound departments—recommended PPCV. Written informed consent was obtained from the patient’s family.

The procedure was performed as follows: First, under ultrasound guidance, the right main branch of the portal vein was successfully punctured percutaneously using an 18G needle. A guidewire (0.89 mm × 150 cm, Terumo Corporation, Japan) was inserted. Under digital subtraction angiography (DSA) guidance, a drainage catheter (8F × 25 cm, Argon Medical Devices, United States) was advanced past the main portal vein tumor thrombus and positioned into the superior mesenteric vein. The distal end of the drainage catheter was coiled into a pigtail shape to ensure stability (Figure 2B, green dashed arrow). The extracorporeal portion of the portal drainage catheter was then connected to the existing subclavian central venous catheter using a sterile standard IV extension set (Figure 2, blue dashed line).

Postoperatively, to prevent thrombosis within the circuit, the patient received a continuous infusion of unfractionated heparin of 12 U/kg/h via the catheter, maintaining the activated partial thromboplastin time (APTT) at 1.5–2.0 times the control value (15).

Direct portal pressure measurements were obtained via the drainage catheter immediately before and 30 min after connection to the central venous line. The pressure dropped significantly from 40.6 mmHg (pre-connection) to 18.8 mmHg (post-connection). Post-procedural monitoring revealed a transient rise in liver enzymes and bilirubin (e.g., AST: 410 U/L, ALT: 105 U/L on 1 December), consistent with post-embolization syndrome. However, these parameters revealed a clear recovery trajectory coinciding with the hemodynamic relief provided by the shunt. By 9 December, key indicators had significantly improved (AST: 99 U/L, TB: 25.3 μmol/L, and albumin: 34.3 g/L), providing biochemical corroboration of the procedural success. Subsequently, pressure was monitored twice daily for 1 week, maintaining a direct portal venous pressure range of 18–28 mmHg (16).

Post-procedure, the patient continued fasting and received symptomatic treatment, including hepatoprotective agents, gastric protection, and human serum albumin. Under continuous heparin flushing, no obvious bloody drainage was observed from the gastric tube within 24 h, nor was there any melena. Hemoglobin levels stabilized. The patient was transferred from the ICU to the general ward after 1 week. His condition stabilized, allowing for discharge 2 weeks post-procedure, at which point the PPCV catheters were removed. Notably, the patient resumed a liquid diet 3 days after PPCV, with no recurrence of hematemesis or melena from the resumption of oral intake until discharge. Serial blood ammonia levels measured at 24 h, 48 h, 72 h, 1 week, and 2 weeks post-operation showed no significant elevation, and liver function did not deteriorate further.

However, 2 months later, the patient succumbed to the progression of his underlying advanced malignancy. The cause of death was multiple organ failure secondary to tumor progression, as the patient and family had declined further anti-tumor therapy (17).

## Discussion

We present a case of a patient with hepatocellular carcinoma (CNLC Stage IIIa) complicated by main portal vein tumor thrombus and decompensated cirrhosis, presenting with recurrent, life-threatening esophagogastric variceal bleeding. Current literature establishes that PVTT is a major predictor of a poor prognosis in HCC (21). By obstructing portal flow, PVTT exacerbates portal hypertension (PH), leading to severe complications such as EGVB, refractory ascites, and portal hypertensive enteropathy (9, 22). These complications not only deteriorate liver function and increase mortality risk (11) but also severely limit the feasibility of subsequent anti-tumor treatments (23).

Although the patient had previously received targeted therapy (lenvatinib and bevacizumab) and immunotherapy (tislelizumab and sintilimab) known to improve survival in BCLC Stage C patients (12, 14), recurrent EGVB necessitated the cessation of these life-prolonging medications, contributing to rapid tumor progression.

While TIPS is generally the gold standard for refractory variceal bleeding due to its efficacy in reducing portal pressure (24, 25) and bridging patients to systemic therapy (10, 18, 25), its application in patients with extensive PVTT (Vp4) or cavernous transformation remains controversial and technically demanding. Although advances in portal vein recanalization (PVR) (26) and transcollateral TIPS (27, 28) have expanded indications, the complete occlusion of the portal vein by tumor thrombus or cavernous transformation in our patient, combined with his hemodynamic instability, made these complex procedures unfeasible. Similarly, transjugular extracorporeal portosystemic shunt (TEPS) requires highly specialized expertise and was not a viable option in this emergency setting (29).

PPCV represents a novel technical innovation developed by our team. To the best of our knowledge, this is the first reported clinical application globally. Mechanistically, PPCV shares the fundamental principle of TIPS: creating a portosystemic shunt to lower portal pressure. However, it offers several distinct advantages in this specific high-risk population: (1) High success rate: Percutaneous ultrasound-guided puncture of the portal vein is technically straightforward and allows for rapid access. In this case, success was achieved on the first attempt. (2) Rapid implementation: By using a pre-existing central venous catheter, the shunt can be established immediately by connecting the external circuits, avoiding the time-consuming steps of internal stent placement. (3) Reduced procedural risk: The technique uses a small puncture tract, minimizing the risk of capsular bleeding or parenchymal injury associated with traditional TIPS, which is critical for hemodynamically unstable patients. (4) Controllability: The extracorporeal nature of the shunt allows for flow regulation. This helps prevent complications associated with sudden hemodynamic shifts, such as overt hepatic encephalopathy. In our case, blood ammonia levels remained stable for 2 weeks. (5) Bridging capability: PPCV provides a crucial window for endoscopic healing or medical stabilization.

Despite its success in this case, PPCV has limitations inherent to its external design: (1) Risk of catheter dislodgment: As an external device, secure fixation is paramount. In this case, meticulous nursing care prevented dislodgment. (2) Thrombotic complications: The long external tubing increases the risk of thrombosis. Continuous heparin flushing was required. Although no active bleeding occurred, catheter thrombosis and blockage were observed during the course, which were successfully managed by catheter exchange and re-insertion. This highlights the necessity of careful balancing between bleeding and clotting risks. (3) Temporary nature: The device was removed after 2 weeks. Data regarding the long-term safety and feasibility of prolonged PPCV use are lacking.

## Conclusion

PPCV has been proven to be a simple, feasible, and effective salvage technique for controlling refractory bleeding in a complex case of HCC with PVTT where TIPS was contraindicated. It successfully reduced portal pressure and achieved hemostasis without immediate complications. While it does not alter the oncological prognosis of advanced HCC, PPCV offers a valuable therapeutic bridge for high-risk patients. Further studies are warranted to refine technical protocols, define optimal indications, and evaluate safety in a larger cohort.

## Data Availability

The raw data supporting the conclusions of this article will be made available by the authors, without undue reservation.
